# Dengue dynamics, predictions, and future increase under changing monsoon climate in India

**DOI:** 10.1038/s41598-025-85437-w

**Published:** 2025-01-21

**Authors:** Yacob Sophia, Mathew Koll Roxy, Raghu Murtugudde, Anand Karipot, Amir Sapkota, Panini Dasgupta, Kalpana Baliwant, Sujata Saunik, Abhiyant Tiwari, Rajib Chattopadhyay, Revati K. Phalkey

**Affiliations:** 1 Centre for Climate Change Research, Indian Institute of Tropical Meteorology, Ministry of Earth Sciences, Pune, 411008 India; 2 Department of Atmospheric and Space Sciences, Savitribai Phule Pune University, Pune, India; 3 Earth System Science Interdisciplinary Center (ESSIC)/DOAS, University of Maryland, College Park, MD USA; 4 Interdisciplinary Program in Climate Studies, Indian Institute of Technology Bombay, Powai, Mumbai, 400076 India; 5 Department of Epidemiology and Biostatistics, School of Public Health, University of Maryland, College Park, MD USA; 6 Future Innovation Institute, Seoul National University, Siheung, Republic of Korea; 7 Health Department, Pune Municipal Corporation, Pune, India; 8 Harvard TH Chan School of Public Health, Boston, MA USA; 9 Government of Maharashtra, Mantralaya, Mumbai India; 10 Natural Resources Defense Council, New Delhi, India; 11 Climate Research and Services, India Meteorological Department, Pune, India; 12 Heidelberg Institute of Global Health, University of Heidelberg, Heidelberg, Germany; 13 Division of Epidemiology and Public Health, University of Nottingham, Nottingham, UK

**Keywords:** Epidemiology, Climate-change impacts, Machine learning, Climate sciences

## Abstract

The global burden of dengue disease is escalating under the influence of climate change, with India contributing a third of the total. The non-linearity and regional heterogeneity inherent in the climate-dengue relationship and the lack of consistent data makes it difficult to make useful predictions for effective disease prevention. The current study investigates these non-linear climate-dengue links in Pune, a dengue hotspot region in India with a monsoonal climate and presents a model framework for predicting both the near-term and future dengue mortalities. Dengue mortality and meteorological conditions over a twelve-year period (2004–2015) are analyzed using statistical tools and machine learning methods. Our findings point to a significant influence of temperature, rainfall, and relative humidity on dengue mortality in Pune, at a time-lag of 2–5 months, providing sufficient lead time for an early warning targeted at curbing dengue outbreaks. We find that moderate rains spread over the summer monsoon season lead to an increase in dengue mortality, whereas heavy rains reduce it through the flushing effect, indicating the links between dengue and monsoon intraseasonal variability. Additionally, warm temperatures above 27°C and humidity levels between 60% and 78% elevate the risk of dengue. Based on these weather-dengue associations, we developed a machine-learning model utilizing the random forest regression algorithm. The dengue model yields a skillful forecast, achieving a statistically significant correlation coefficient of *r* = 0.77 and a relatively low Normalized Root Mean Squared Error score of 0.52 between actual and predicted dengue mortalities, at a lead time of two months. The model finds that the relative contributions of temperature, rainfall, and relative humidity to dengue mortality in Pune are 41%, 39%, and 20%, respectively. We use the dengue model in conjunction with the climate change simulations from the Coupled Model Intercomparison Project phase 6 for the future dengue mortality projections under a global warming scenario. In a changing climate, dengue-related mortality in Pune is projected to rise by 13% in the near future (2021–2040), 23–40% in the mid-century (2041–2060), and 30–112% in the late century (2081–2100) under low-to-high emission pathways in response to the associated increase in temperature and changes in monsoon rainfall patterns.

**Supplementary Information:**

The online version contains supplementary material available at 10.1038/s41598-025-85437-w.

## Introduction

Dengue has emerged as the world’s most widespread and rapidly increasing vector-borne disease, with nearly half of the global population now at risk. India accounts for a third of the global dengue disease burden, out of an annual global incidence of 100–400 million infections^1,2^. Dengue is a mosquito-borne viral disease transmitted primarily by female *Aedes* mosquitoes, particularly *Aedes aegypti* and to a lesser extent, *Aedes albopictus*, when they feed on human hosts. According to data from the National Center for Vector Borne Diseases Control (NCVBDC), dengue cases in India have quadrupled from 2015 to 2020^3^.

Dengue fever is a climate-sensitive disease and is significantly influenced by changes in meteorological conditions. Different studies in various regions have reported vector-borne epidemics with climatic drivers such as temperature, precipitation, relative humidity, wind velocity, and climate modes such as the El Niño-Southern Oscillation (ENSO)^4–10^. Temperature and humidity impact dengue incidence by affecting larvae development, adult feeding behavior, and mosquito survival^11–15^. Rainfall, on the other hand, provides habitats for the aquatic stages of mosquitoes. While mosquitoes require sufficient rainfall for breeding and larval development^12,15,16^, excessive rainfall can cause breeding sites to overflow, disrupting mosquito breeding and destroying developing larvae^17,18^. This phenomenon, known as rainfall flushes, has been observed in both experimental and field settings^9,10^. Associations between rainfall and dengue incidence vary widely from weak or no connection^19,20^ to as much as a 21% increase in dengue incidence in response to increased rainfall^21^. This indicates that there is a large regional and location-specific environmental dependence of the rainfall-dengue relationship.

According to the sixth assessment report of the Intergovernmental Panel on Climate Change (IPCC), the burden of dengue may change globally, nationally, and locally in response to the continuous increase in global surface temperature and changes in rainfall patterns^22^. Additionally, studies suggest that climate change is expanding the geographic spread of some vectors and vector-borne diseases, including malaria and dengue fever, to higher altitudes and latitudes^23,24^.

Vector control operations through breeding source reduction remain the only efficient method to stop dengue transmission. Dengue early warning systems can help authorities in proactive measures to prevent and manage dengue outbreaks. In India, the India Meteorological Department publishes a ‘climate information for health’ report based on the Global Forecast System (GFS) and the Extended Range Forecast System (ERFS) for the transmission windows of temperature conducive to the development of vector-borne diseases such as malaria and dengue^25^. This is a temperature-driven generalized model for India that overlooks other potential climate-based predictors like humidity and rainfall patterns. Despite the evident links between climate and dengue, we lack a comprehensive and multivariate framework for dengue predictions and projections.

**Fig. 1:**
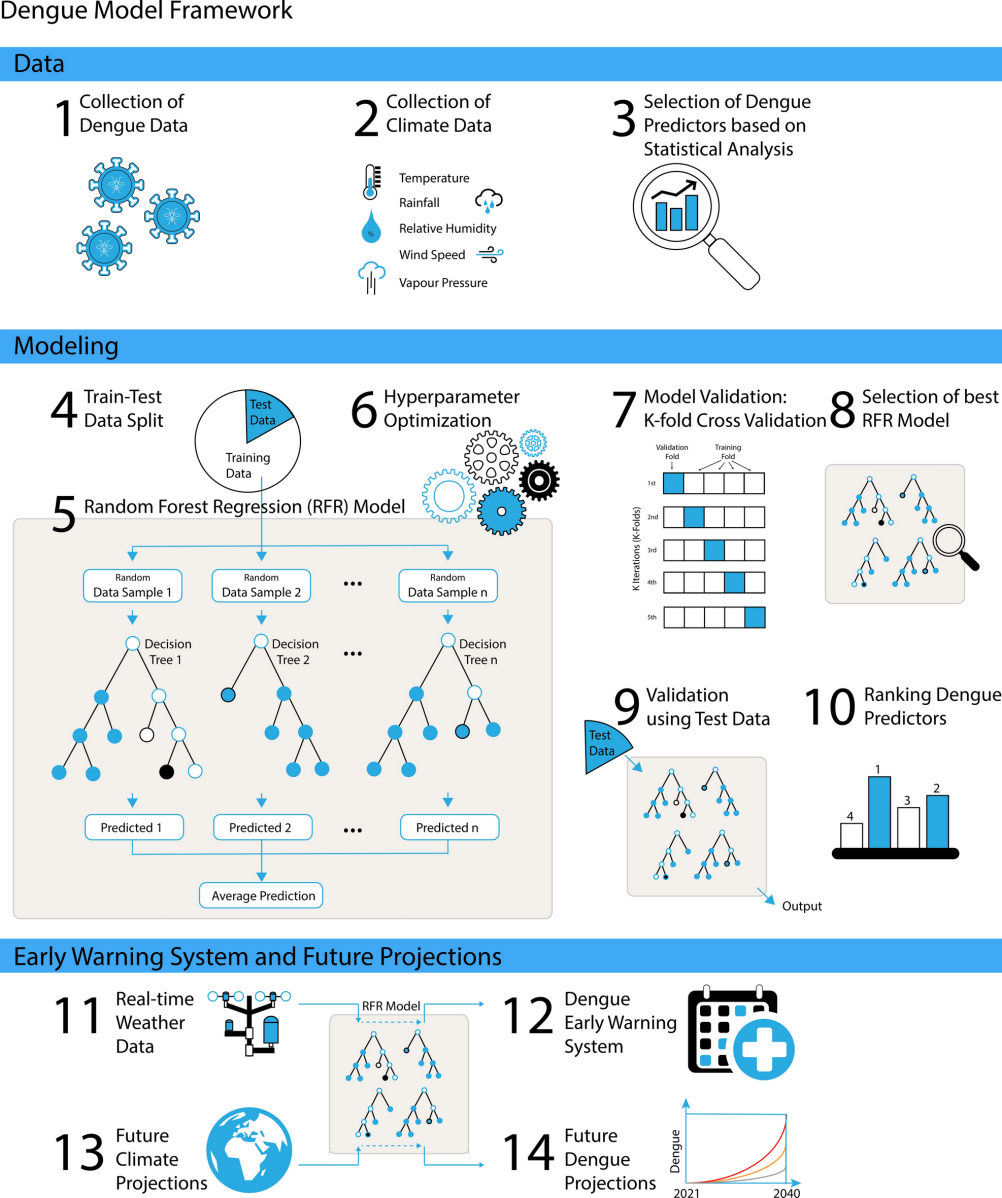
The dengue model framework here illustrates the step-by-step processes involved in developing an early warning system and future projections of dengue mortality. The processes include collecting both dengue and meteorological data (steps 1 and 2), selecting relevant dengue predictors through statistical analysis (step 3), and developing a climate-based Random Forest Regression (RFR) model for dengue predictions. Model development includes steps such as test-train splitting, fitting the model to training data, hyperparameter optimization, k-fold cross-validation, model validation using test data, and variable importance ranking (steps 4–10). The resulting RFR model can serve as an early warning system with real-time inputs (steps 11 and 12) and can also be used to prepare future dengue projections using the climate change projections from the CMIP6 models (steps 13 and 14). This framework provides a comprehensive approach for understanding the impacts of climate change on dengue in Pune and can be used as a template for similar studies in other regions.

A dengue early warning system that incorporates all potential climate-based dengue predictors and their combined interactions with dengue at a regional scale is the need of the hour in an environment where climate change is projected to exacerbate vector-borne diseases like dengue. The current study explores the climate-dengue associations in urban Pune, a dengue hotspot in the state of Maharashtra in India with a high dengue disease burden and devises a dengue model framework for early warnings and future projections of the disease (Fig. 1). This framework provides a comprehensive approach for understanding the impacts of climate change on dengue in Pune and can be used as a template for similar studies in other regions. Region-specific future projections of dengue can significantly enhance prevention strategies and inform policymaking, ultimately aiding in the mitigation of disease outbreaks in a warming climate.

## Results

**Fig. 2:**
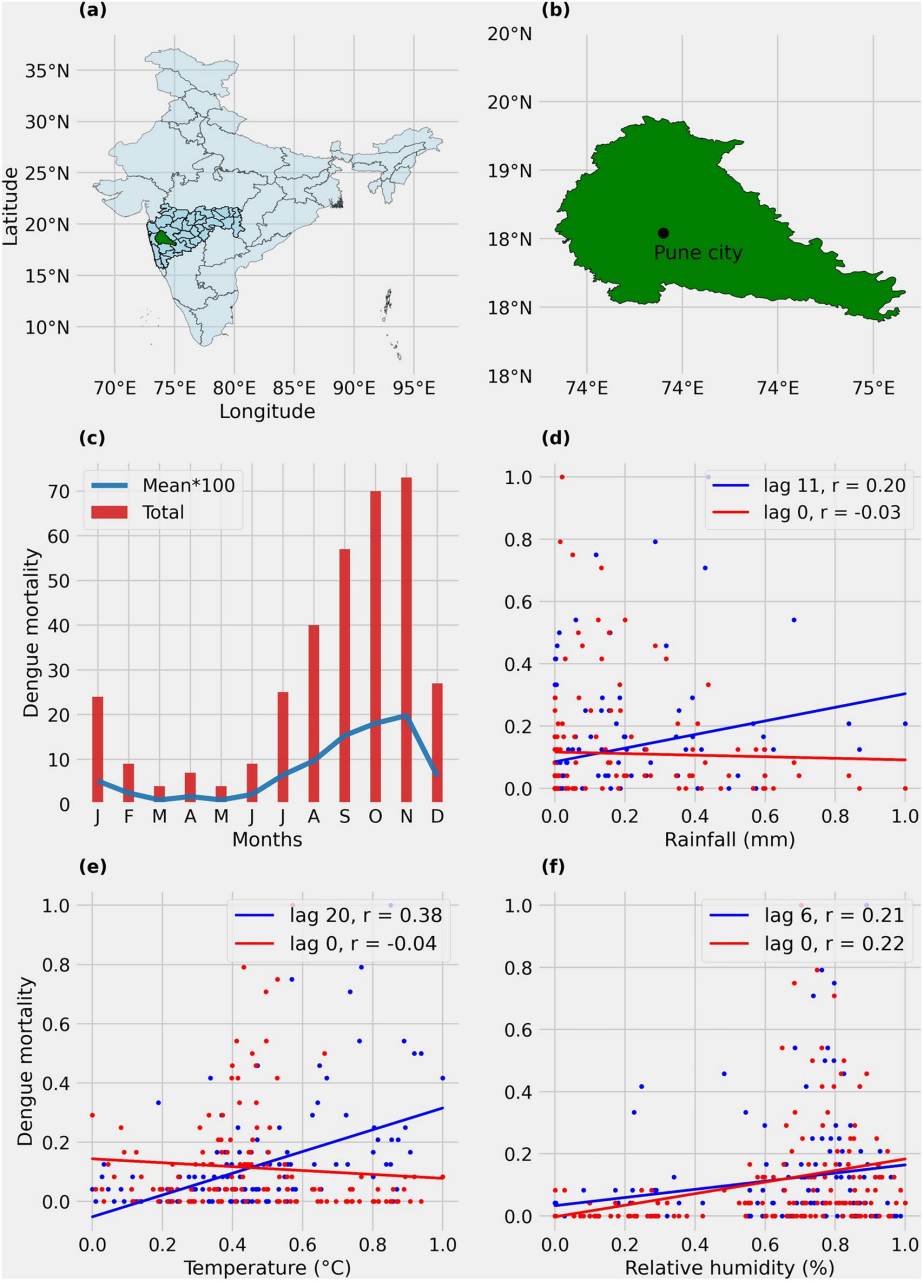
(**a**) Geographical details of Pune district in Maharashtra, India. (**b**) Detailed view of Pune district highlighted in green. (**c**) Monthly cumulative (red bars) and mean (blue line) dengue mortalities over Pune from 2004 to 2015. Scatter plot (round markers) and linear fit (line plot) between normalized monthly mean values of (**d**) rainfall, (**e**) temperature, and (**f**) relative humidity on the x-axis and dengue mortalities over Pune on the y-axis. Data points are shown for zero lag in red and at the lag with maximum correlation in blue. Temperature, rainfall, and relative humidity show a statistically significant (*p* < 0.05) correlation of 0.38, 0.20, and 0.21 with dengue mortality at a lag of twenty weeks, eleven weeks, and six weeks, respectively.

Dengue mortality over Pune shows a clear seasonality during the study period (2004–2015) (Fig. 2c). It is low from December to May but begins to rise after the onset of the summer monsoon rainfall in June and peaks in November. The maximum number of dengue mortalities reported over Pune during the study period was 81 in the year 2014.

### Climatic factors affecting dengue mortality in Pune

By incorporating insights from the previous studies and establishing defined thresholds based on statistical analysis, we identified favorable windows of climatic conditions for increased dengue mortalities over Pune and devised a novel dengue metric for a preliminary statistical analysis. The novelty of our metric is that instead of looking into the direct relationship between climate and dengue, the number of days or weeks with favorable climatic conditions are considered here. Also, unlike existing studies examining the rainfall-dengue association, this metric employs three rainfall dependent variables—wet weeks, flush counts, and monsoon intraseasonal oscillations—to understand how rainfall patterns impact dengue mortality in Pune. The various climate-based predictors included in the metric are described in detail in the following sections (sections “Temperature” to “Humidity”).

#### Temperature

Mosquitoes, being poikilotherms, undergo fluctuations in their internal temperature depending on the surrounding environment. They face various risks, including desiccation (extreme drying), changes in metabolism, and reduced mobility due to thermal variations^26^. The mean temperature range of 27–35 °C is found to be optimal for increased dengue transmission considering several vector parameters like longevity, fecundity (the number of eggs deposited by each female vector), parity (the number of times a female has laid eggs), gonotrophic cycle (the time between a blood meal and oviposition), extrinsic incubation period (the time it takes for the virus to develop inside the mosquito), and intrinsic incubation period (time from human infection to symptom onset)^12,27–30^. However, it is important to verify if this temperature range favors dengue mortality in Pune as studies have shown that the optimal temperature for dengue transmission vary based on local climate conditions^31,32^. For instance, in Delhi, dengue transmission was positively correlated with higher temperatures, even in the range of 30–39 °C^31^. In contrast, the highest mean temperature recorded in Pune during the study period (2004–2015) was 35 °C, suggesting that the identified optimal temperature range (27–35 °C) is specific to Pune’s climate. As shown in Supplementary Fig. S1-a, we find a statistically significant positive correlation (*r* = 0.70, *p* < 0.05) between the annual dengue mortality and the number of days with optimal temperatures (27–35 °C) during the summer monsoon season (June to September) in Pune. This temperature window is specific to Pune and maybe different for other regions. Thus, rather than looking at the direct relationship between dengue and temperature, the number of days with favorable conditions for dengue vector and virus yields a more meaningful result.

#### Rainfall flushes and wet weeks

To better understand the rainfall-dengue relationship, we introduced rainfall-dependent variables, such as wet weeks, and rainfall flushes for Pune. Wet weeks are defined as weeks with a minimum of 0.5 mm and a maximum of 150 mm of rainfall, and when the weekly cumulative rains exceed 150 mm, such events are termed rainfall flushes. The current study reveals that weekly cumulative rains between 0.5 mm and 150 mm lead to an increase in dengue mortality (*r* = 0.85, *p* < 0.05) (Supplementary Fig. S1-b, Table 1), while rains above that threshold reduce dengue mortality (*r* = − 0.77, *p* < 0.05) in Pune through the flushing effect (Supplementary Fig. S1-c, Table 1). Rainfall thresholds that are used to define flushing can vary depending on the region^33,34^. Various weekly rainfall thresholds were examined to identify the one that exhibits the strongest correlation with dengue mortality in Pune. The current study recommends using a weekly threshold to define wet weeks and rainfall flushes, considering the cumulative effect of rainfall reaching the threshold within a week.

**Table 1:** Comparison of years with high (2010, 2014, 2015) and low dengue mortalities (2008, 2011, 2013) in Pune (2004–2015) based on dengue metric variables, wet weeks, flush count, count of active and break days of monsoon, and count of optimal temperature days. High dengue years in Pune (columns represented in red shade) exhibit lower cumulative rainfall, fewer rainfall flushes, and active-break days but a higher count of wet weeks compared to years with low dengue mortality (columns represented in blue shade). High dengue years have more days with optimal mean temperature (27–35 °C).

	High dengue count years			Low dengue count years		
Year	2010	2014	2015	2008	2011	2013
Deaths	68	81	64	13	14	16
Total rainfall (mm)	1247	1480	1447	2985	2305	2069
No. of wet weeks	37	33	32	20	25	26
Flush count (> 150 mm)	1	2	2	8	6	6
Count of active days	13	17	18	52	49	41
Count of break days	9	28	21	44	50	27
Count of days with optimal temperature (27–35 °C)	107	140	145	99	93	91

#### Monsoon intraseasonal oscillations

**Fig. 3:**
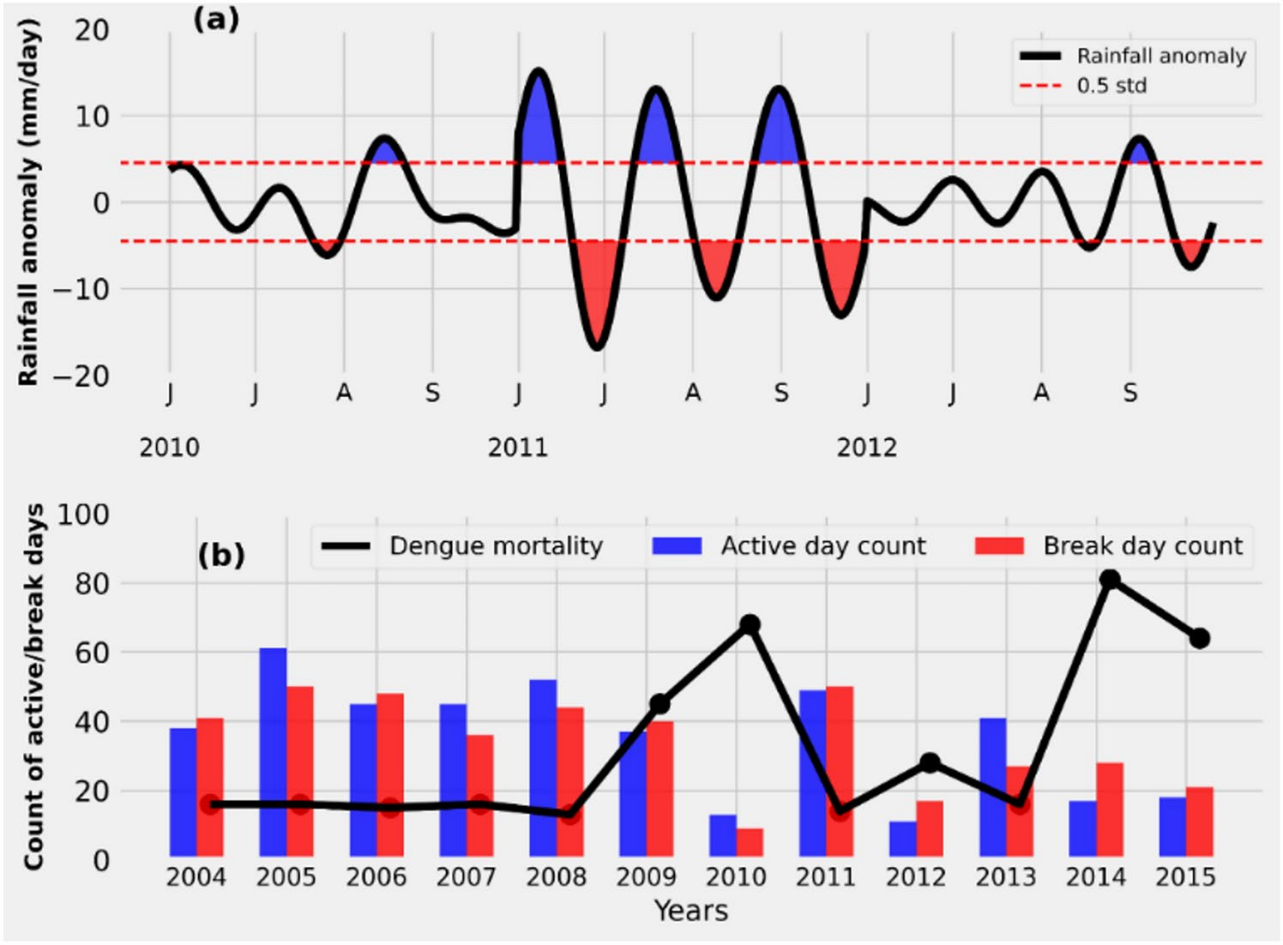
(**a**) Rainfall anomaly timeseries filtered for its intraseasonal oscillations during 2010–2012 (note that this is for a sample period, and the entire filtered time series is not shown here). Active (blue shaded) and break days (red shaded) are those with rainfall anomalies greater than 0.5 standard deviations (dashed red line) and less than –0.5 standard deviations, respectively, of the summer monsoon rainfall anomaly (June–September) from 1995–2015. (**b**) Count of active (blue bars), break days (red bars), and dengue mortality (black line) from 2004 to 2015 over Pune. The number of active and break days is lower in high dengue mortality years (2010, 2014, 2015) compared to other years with low dengue mortality.

The Indian summer monsoon rainfall exhibits strong variability at intraseasonal timescales, known as monsoon intraseasonal oscillations, characterized by active (wet) and break (dry) phases of the monsoon^35,36^. Monsoon intraseasonal oscillations are obtained using bandpass filtering for the dominant intraseasonal timescale (30–90 days) (Fig. 3a)^37–39^. We consider active days as those with rainfall anomalies exceeding 0.5 standard deviations of the summer monsoon (June to September) rainfall anomaly of the baseline period from 1995–2015, while break days are defined as those with rainfall anomalies less than − 0.5 standard deviations. As demonstrated in Fig. 3b and Table 1, the years reporting high dengue mortality in Pune (2010, 2014, and 2015) are characterized by a lower number of active and break days. Conversely, the years with low dengue mortality (2008, 2011, and 2013) are characterized by a higher number of active and break days (*r* = − 0.74, *p* < 0.05). Currently, the India Meteorological Department provides extended-range forecasts with information on the active-break cycles of monsoon, 10–30 days in advance for the entire country. Utilizing these forecasts can offer additional lead time for dengue predictions. Thus, monsoon intraseasonal oscillations could serve as a valuable predictor of dengue with improved skills in ISMR predictions.

The rainfall-dependent variables presented here highlight clear differences in the patterns of rainfall during high and low dengue years in Pune. As shown in Table 1, high dengue years in Pune have fewer rainfall flushes, more wet weeks, and fewer active-break days. An increased number of wet weeks, along with fewer rainfall flushes, indicates that rainfall is more evenly spread over time, reducing the likelihood of mosquito eggs and larvae being washed away. Fewer active-break days also suggest that the variability in monsoon rainfall is lower during high dengue years. Thus, years with high dengue mortality in Pune are associated with moderate rainfall distributed over time.

Conversely, low dengue years are linked to more rainfall flushes and fewer wet weeks (Table 1). Although these years have higher total rainfall, the rains are extreme, which can flush out mosquito eggs and larvae. Additionally, the increased number of active-break days suggests greater variability in rainfall during low dengue years, with more number of extreme rainfall events. This pattern contrasts with the moderate, evenly distributed rains that favor dengue transmission.

In summary, our findings suggest that it is not the cumulative amount of rainfall, but rather the pattern of rainfall, that plays a crucial role in influencing dengue transmission in Pune. Moderate rains spread over time creates favorable conditions for dengue, whereas extreme rainfall events reduce the dengue risk through their flushing effect.

#### June cumulative rains

Major rain-bearing months in Pune are from June to September, and more than 80% of dengue mortalities in the district occur after the onset of monsoon in June (Fig. 2c). Studies have shown that mosquito eggs can survive drying out for up to eight months, implying that eggs laid after the monsoon season in the preceding year may rely on the June rains of the current year for survival^15,40^. However, heavy rains in June can wash out those eggs and play an important role in reducing dengue-related mortalities for the entire year by controlling the vector population. We find a statistically significant negative association (*r* = − 0.66, *p* < 0.05) between annual dengue mortality and the June cumulative rains in the corresponding year in Pune during the study period (2004–2015) and is more evident in recent years with high dengue mortality (Supplementary Fig. S1-d). Conversely, we find no statistically significant correlations (*p* < 0.05) between cumulative rainfall in the other monsoon months and annual dengue mortality (Supplementary Fig. S7, Table S3).

However, it’s important to note that all monsoon months significantly contribute to mosquito breeding and dengue incidence and the other rainfall-dependent variables in the dengue metric, such as wet weeks and flush counts, are calculated based on rainfall throughout the entire year.

#### Humidity

Relative humidity is found to favor the percentage of hatching, survival rate, and biting frequency of adult dengue mosquitoes and allows the infected female mosquitoes to complete more than one replication cycle of the virus^16,41^. A minimum of 60% relative humidity is required for *Ae. aegypti* mosquitoes to survive because low humidity causes water to evaporate from the mosquito’s body and dries out their body fluids^42^. Also, the mosquitoes will have a short lifespan below 60% humidity and will not be able to transfer the virus from its stomach to salivary glands, which prevents them from becoming vectors^43^. Conversely, an increase in relative humidity beyond a specific threshold can result in a decrease in dengue incidence^44,45^. This is a critical environmental parameter for Pune where the humidity shows high variability across the seasons. According to our analysis, dengue mortality over Pune is high when relative humidity levels are between 60 and 78% during the summer monsoon season (June to September) (*r* = 0.72, *p* < 0.05). This range closely aligns with findings from a study in Delhi, India, where a relative humidity of 70–80% was identified as ideal for *Ae. aegypti* mosquitoes^46^. Additionally, studies from Guangzhou, China—another monsoonal region—reported similar upper thresholds of 76% and 79% for relative humidity favoring increased dengue incidences^32,45^. Like temperature and precipitation, relative humidity also exhibits a non-linear relationship with dengue (Supplementary Fig. S1-e).

In conclusion, our analysis of observed dengue mortality and meteorological variables from 2004 to 2015 in Pune demonstrates that moderate rainfall distributed over time contributes to an increase in dengue mortality, while heavy rains have the potential to reduce it through flushing effect. Furthermore, our findings indicate that years with higher dengue mortality rates in Pune are characterized by temperatures ranging from 27 °C to 35 °C and relative humidity levels between 60 and 78% during the Indian summer monsoon season. The dengue metric presented here is part of the preliminary statistical analysis aimed at understanding the climatic conditions that favor dengue mortality in Pune and is based on the basic linear correlation analysis. However, the climate-dengue associations are complex due to the non-linear and compound interactions between potential predictor variables. Therefore, we developed a machine learning model to better capture and analyze these intricate non-linear associations.

### Dengue early warning system for Pune

Machine learning methods are found to be superior to traditional statistical methods in understanding and predicting the complex interactions between multiple variables^47^. To identify potential climatic variables that influence dengue incidence in Pune and to understand their cumulative effect, we used the machine learning technique of Random Forest Regression (RFR) on 12 years of observed dengue mortality and meteorological data (2004–2015) at weekly timescales. Random Forest is an ensemble learning method that combines the predictions of multiple decision trees to create a more robust and accurate model^48^. A combination of individual models like this can help to reduce overfitting and improve generalization.

Despite the availability of many advanced machine learning algorithms, RFR was selected for this study due to its favorable prediction performance in prior research^49–51^ and its relative ease of comprehension, making it suitable for researchers new to machine learning. RFR is highly flexible and can handle high-dimensional data with non-linear effects and numerous covariate interactions. Its built-in mechanism to rank significant predictors also provides valuable insights into the model predictions^50^. Predictor inputs used in the RFR model (dengue model from here) are weekly means of temperature, cumulative rainfall, relative humidity, and weekly count of active and break days. These weekly predictors were chosen from a larger group of variables based on statistical analyses and the improvement in model skill (Fig. 1, steps 2 and 3). The other derivative variables from the dengue metric (section “Climatic factors affecting dengue mortality in Pune”) were not used as additional individual predictors in the dengue model, as their inclusion did not enhance model skill. This is because RFR is a decision tree-based algorithm, capable of capturing the non-linear associations between predictors and the target^52^. It can identify the optimal climatic conditions for increased dengue risk using basic climate variables such as mean temperature, cumulative rainfall, and mean relative humidity, without the need to explicitly include derived variables like the count of days with optimal temperature, wet weeks, or flush counts. Although the dengue metric variables were not directly used in the model, they aid in interpreting the predictions and projections generated by the dengue model.

It is critical to incorporate the lag between favorable weather conditions and dengue mortality into the dengue model, as it accounts for multiple processes occurring over different time spans, from mosquito breeding to human host getting infected with the dengue virus. The mosquito’s lifecycle from an egg to an adult, takes about 8–10 days, while the extrinsic incubation period of the dengue virus inside an infected mosquito takes 5–33 days^12^. After infecting a human through a mosquito bite, the virus has an intrinsic incubation period of about 2–15 days in the human body^53^. A person typically develops viremia, a condition characterized by a high level of the dengue virus in the blood, four days after being bitten by an infected *Ae. aegypti* mosquito. Viremia can last up to 12 days but usually lasts around five days^54^. Studies report that the possible lag between hospital admission (after the onset of symptoms) and dengue mortality ranges from 3–15 days^55,56^. Altogether, from the availability of aquatic habitats for mosquito breeding to a dengue mortality, it takes approximately 1–3 months (8–73 days). In addition to this, the dengue vectors capable of transmitting dengue virus can survive for about 30 days^21^.

Similar to the methodology followed in the prior studies on dengue modeling using climatic variables, input variables are provided to the dengue model at the lags where variable-dengue correlations are strongest^57,58^. Using cross-correlation analysis, our study finds that dengue mortality over Pune (2004–2015) shows maximum correlation with mean temperature (*r* = 0.38, *p* < 0.05) and cumulative rainfall (*r* = 0.20, *p* < 0.05) at lags of 20 and 11 weeks, respectively (Fig. 2d,e). Relative humidity shows a statistically significant correlation with dengue mortality at a lag of six weeks (*r* = 0.21, *p* < 0.05), with a slightly stronger correlation at lag zero (*r* = 0.22, *p* < 0.05) (Fig. 2f). Though relative humidity at lag zero had a slightly stronger correlation with dengue mortality, relative humidity at a lag of six weeks was chosen for the dengue modeling due to its potential for longer-lead predictions. The dengue model achieved highest prediction skill by using temperature twenty weeks prior, rainfall eleven weeks prior, and relative humidity six weeks prior for predicting dengue mortality of the current week.

It is important to note that these linear correlation values are part of the preliminary statistical analysis aimed at identifying the lag in climate-dengue association (Fig. 2d–f) and do not represent the predictive power of each variable in forecasting dengue mortality. Given the non-linear and complex nature of climate-dengue interactions, we further developed a machine learning-based dengue model using the random forest regression algorithm to capture the intricate relationships between climatic variables and dengue.

**Fig. 4:**
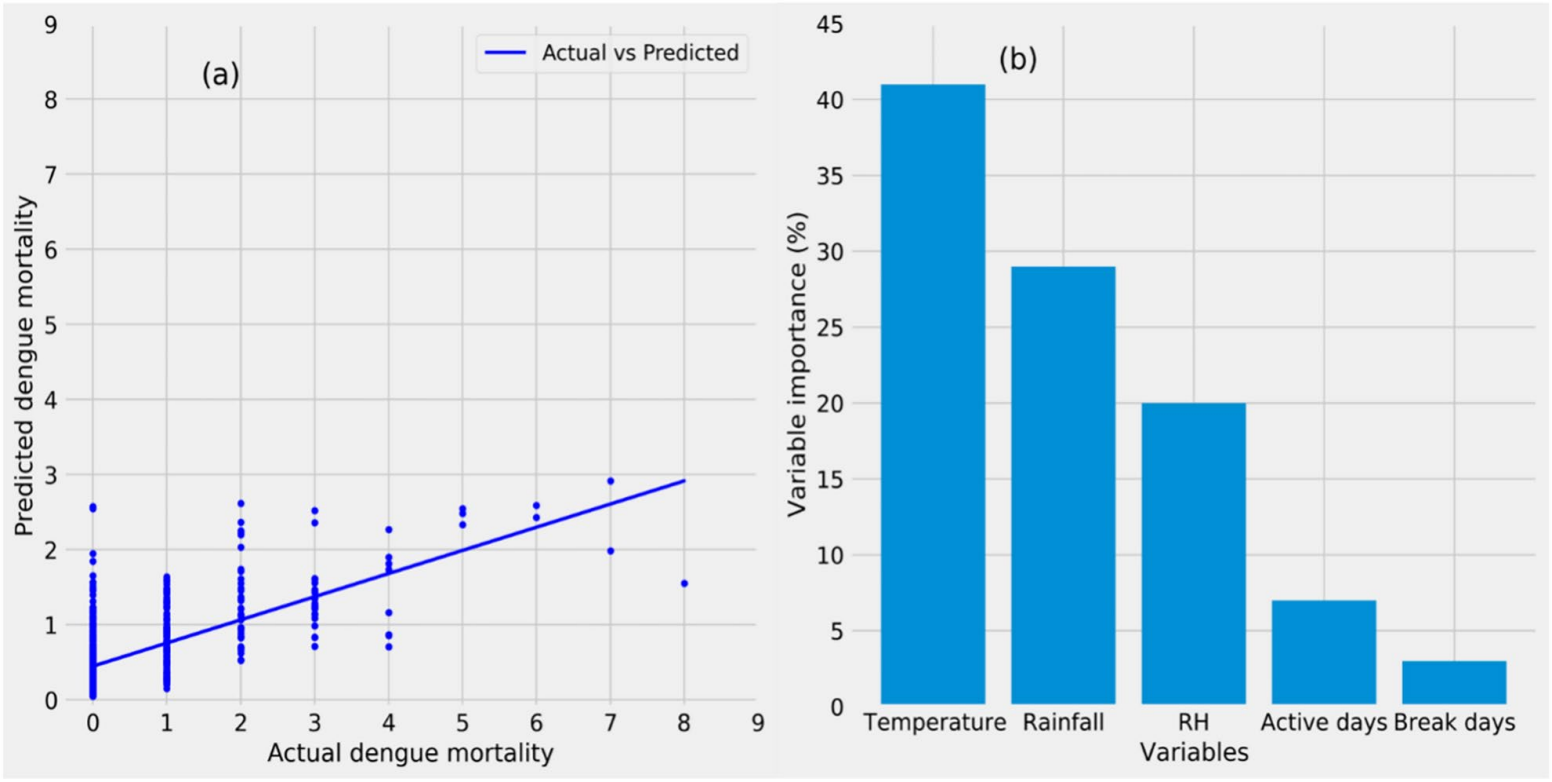
(**a**) Comparison of weekly cumulative dengue mortalities in Pune (x-axis) and the corresponding mortality predictions from the dengue model (y-axis) during the test period 2004 and 2015. The model shows good prediction skill, with a statistically significant correlation coefficient of *r* = 0.77 (*p* < 0.05) and a low normalized root mean squared error (NRMSE) of 0.52 between actual and predicted dengue mortalities. These results indicate that the model can effectively predict dengue mortality in Pune. (**b**) Relative importance of each predictor variable in predicting the dengue mortality in Pune (variable importance). The variable importance of temperature, rainfall, relative humidity, active days, and break days are 41%, 29%, 20%, 7%, and 3%, respectively. Monsoon rainfall and its variability together account for 39% of the variable importance.

We used the meteorological and dengue mortality data from 2005–2014 for training and the years 2004 and 2015 for testing the dengue model. At the end of the optimization of model parameters using *RandomizedSearchCV* and *K-fold cross-validation* (Fig. 1, steps 6 and 7), the model yields a good prediction skill with a statistically significant correlation coefficient of *r* = 0.77 (*p* < 0.05) and a low Normalized Root Mean Squared Error (NRMSE) score of 0.52 between the actual and predicted dengue mortalities during the test period, indicating that the model can effectively predict dengue mortality in Pune (Fig. 4a).

To understand the role of different weather variables in predicting Pune’s dengue mortality, we utilized the variable importance feature of Random Forest algorithms. Variable importance refers to the degree of association between a given predictor variable and the model predictions. From the observed climate and dengue mortality data, the RFR model finds that mean temperature and relative humidity has a variable importance of 41% and 20%, respectively, in predicting dengue mortality over Pune (Fig. 4b). Rainfall and its variability together account for 39% of variable importance, with cumulative rainfall contributing 29% and monsoon intraseasonal variability (active-break days) contributing 10%. The variable importance is directly linked to the model skill, indicating that 41% of the model’s prediction skill is driven by mean temperature and 29% by cumulative rainfall.

### Dengue future projections for Pune

**Fig. 5:**
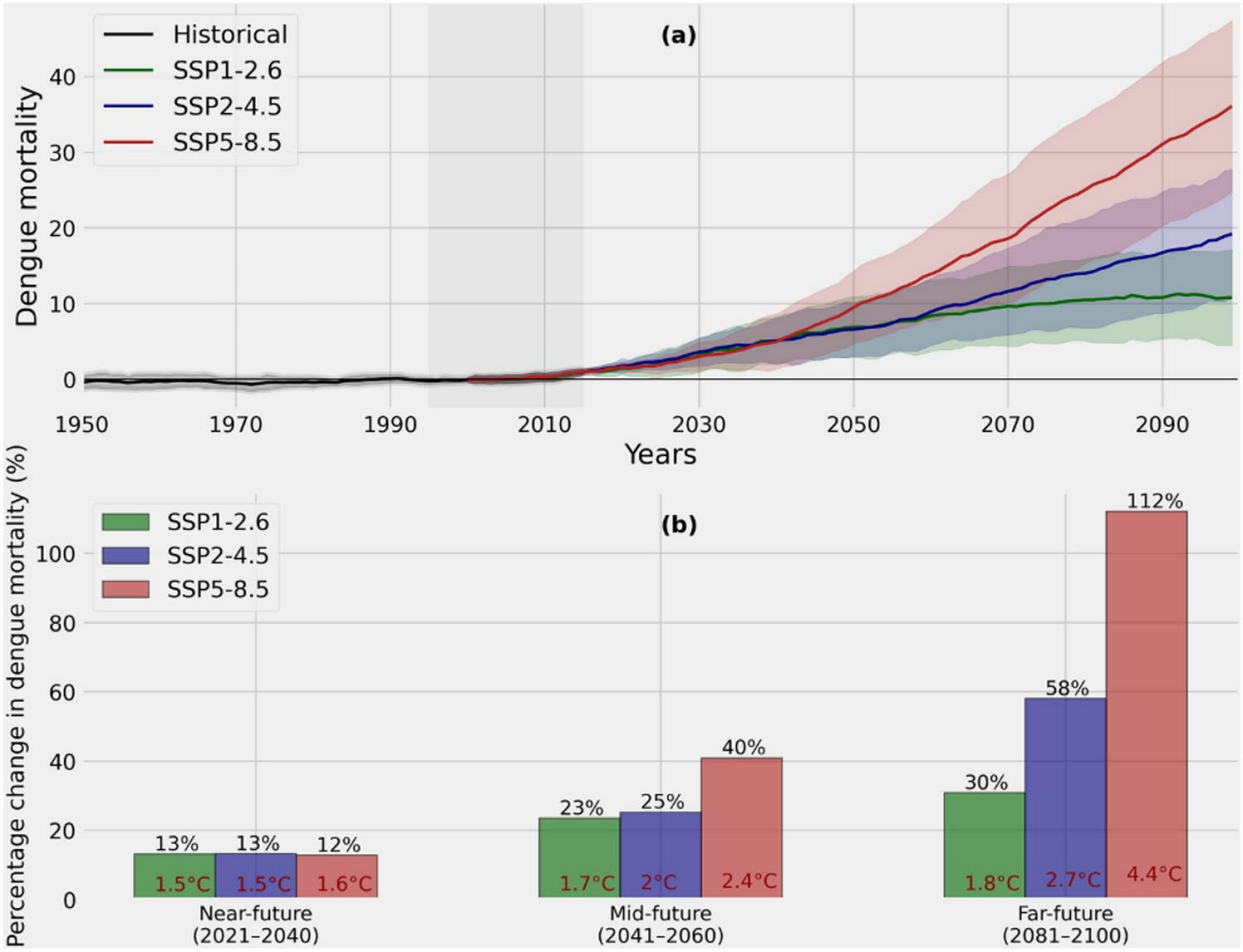
(**a**) The dengue mortality predictions for Pune, based on the dengue model developed for Pune (1950–2100) for the historical (1950–2014) and the future emission pathways (2015–2100, SSP1, SSP2, and SSP5) relative to the reference period of 1995–2014 (light-grey bar). The multi-model ensemble means were computed using a 20-year smoothed time series. Shaded regions in the time series indicate the range of uncertainty or variability, represented by one standard deviation of dengue mortality projections across selected models. (**b**) Projected percentage changes in dengue mortalities over Pune in the near future (2021–2040), mid-century (2041–2060), and late century (2081–2100) emission pathways, relative to the reference period (1995–2014). Changes in average global surface temperature, relative to the pre-industrial period (1850–1900), are indicated in red font within the bars corresponding to the three emission pathways for near future, mid-century, and late century.

Monsoon conditions, particularly rainfall patterns, are projected to change in the coming years and decades over India^59^. This may have significant implications for Pune since the dengue mortality in the region exhibits a strong dependence on the rainfall pattern. The dengue model developed based on the observed climate-dengue associations in Pune is used in conjunction with climate change projections from the Coupled Model Intercomparison Project phase 6 (CMIP6) models for depicting future projections of dengue mortality in the district (Fig. 5a). Dengue mortality in Pune is projected under three distinct Shared Socioeconomic Pathways (SSPs), representing different levels of socioeconomic development and greenhouse gas emissions. SSP1, SSP2 and SSP5 correspond to low, intermediate, and high emissions, respectively. Eight CMIP6 models that best represent the interannual variability in the intraseasonal oscillations of the Indian summer monsoon and spatial distribution of the mean precipitation over India are selected for assessing the future projections of climate and dengue in Pune (Supplementary Table S1). All climatic predictors of the dengue in Pune were computed from the CMIP6 model outputs and provided as inputs into the dengue model at optimal lags to obtain the dengue future projections for the region.

According to the future projections obtained from the dengue model, dengue mortalities over Pune are projected to rise in the future under all emission pathways (SSPs) (Fig. 5a,b). In the near future (2020–40), regardless of the emission pathways, a 12–13% increase in dengue mortalities is expected relative to the reference period (1995–2014). Whereas in the mid-century (2040–60), dengue mortality over Pune is projected to increase by 25% under SSP2 and 40% under SSP5 relative to the reference period (1995–2014) (Fig. 5b). This increase is amplified in the late century (2081–2100), with projections of up to a 112% increase in dengue mortality by 2100 under SSP5. In the late century, the percentage increase in dengue mortality under SSP1 and SSP2 is 30% and 58%, respectively (Fig. 5b). The first virologically proven dengue epidemic in India occurred in Calcutta and the Eastern Coast of India in 1963–1964^60,61^, and hence the future projections are considered from 1950 onwards.

In Fig. 5b, the changes in average global surface temperature compared to the pre-industrial era (1850–1900) are indicated in red font within the bars corresponding to SSP1, SSP2, and SSP5 pathways for the near future, mid-century, and late century time periods. If future emissions can be controlled to keep the global average surface temperature changes below 1.5 °C, the anticipated rise in dengue mortalities relative to the reference period will be below 13%. However, if emissions continue to increase and temperatures exceed the 1.5 °C threshold, the corresponding increase in dengue mortality ranges from 23–40% (Fig. 5b). Alternatively, when the global surface temperature crosses the 2 °C threshold, dengue mortalities are projected to increase by 40–112% relative to the reference period.

### Climatic changes leading to the projected increase in dengue in Pune

To understand the climatic changes leading to the projected increase in dengue mortalities over Pune (Fig. 5a,b), we analyzed the future projections of environmental predictors of dengue in Pune. The future projections from the selected CMIP6 models show that all environmental predictors of dengue are expected to increase in the future (relative to historical levels), with greenhouse gas emissions and socio-economic pathways having a particularly pronounced effect on future projections towards the late century (2081–2100) (Supplementary Fig. S3). In the near-term, the meteorological variables show little change relative to their respective reference period (1995–2014) values. However, by the mid-century, the mean temperature is likely to increase by 1–1.5 °C, cumulative rainfall by 23–27 cm, relative humidity by 0.4–1%, and the number of active-break days by 1–3 days from their corresponding reference period values (Supplementary Table S2). In the far-future, selected CMIP6 models indicate an increase in mean temperature, cumulative rainfall, mean relative humidity, count of active and break days in the range of 1.2–3.5 °C, 28–65 cm, 0.2–1.6%, and 2–5 days, respectively, under the three emission pathways considered in this study. The lower limit in the ranges corresponds to the low emission scenario (SSP1), while the maximum changes are projected under the higher emission scenario (SSP5).

The future trends (2015–2100) in the climatic predictors of dengue mortality in Pune is shown in Table 2. Both temperature and rainfall demonstrate a statistically significant increasing trend across all emission pathways. The count of active-break days shows a statistically significant increasing trend only under SSP2 and SSP5. Additionally, relative humidity exhibits an increasing trend only in the high-emission scenario, SSP5.

**Table 2:** Future trends (per century) in meteorological predictors of dengue mortality in Pune from 2015 to 2100 under the future Shared Socioeconomic Pathways (SSP), (a) SSP1 (sustainability pathway with low emission scenario), (b) SSP2 (middle-of-the-road pathway with intermediate emission scenario), and (c) SSP5 (fossil-fuel intensive pathway with high emission scenario). Trend values are based on the ensemble mean of eight selected CMIP6 models. Statistically significant trends (*p* < 0.05) are marked with an asterisk (*).

Variables	Trend/century		
	SSP1	SSP2	SSP5
Temperature (°C/century)	1.26*	2.3*	4.6*
Rainfall (mm/century)	1.61*	8.7*	15.3*
Relative humidity (%/century)	− 0.3	0.2	1.06*
Active days (days/century)	1.5	3.8*	7.03*
Break days (days/century)	1.1	3.2*	9.01*

Even though all meteorological predictors of dengue mortality in Pune are projected to increase in the future, their collective impact on dengue mortality cannot be determined through linear analysis alone. The dengue model was used to gain insights into the combined effect of projected changes in dengue predictors on dengue mortality in Pune. To isolate the impact of these trends in the predictor variables on dengue mortality projections, we performed experiments detrending the climatic predictors in the dengue model. In these experiments, each predictor variable was detrended individually while keeping the other predictors unchanged. The results of these experiments are presented in Supplementary Figs. S4 and S5. According to these experiments, the trend in the mean temperature has a significant influence, contributing to a 12–22% increase in dengue mortality across low-to-high emission scenarios (Supplementary Fig. S5). In contrast, the trend in rainfall offsets dengue mortality by 2–4% (Supplementary Fig. S5). The percentage changes are calculated for the detrending experiments relative to the original dengue mortality projections. The trends in other variables have little effect on the dengue mortality in Pune.

Our analysis of observed meteorological variables and reported dengue mortalities from 2004 to 2015 in Pune (presented in section “Climatic factors affecting dengue mortality in Pune”) reveals a complex relationship between rainfall patterns and dengue mortality. Specifically, we found that moderate rains spread in time lead to increased dengue mortality over Pune, whereas heavy rains reduce it through the flushing effect (Table 1). To further investigate if the dengue model is picking up this relationship while giving future projections, we performed an experiment in which we modified the rainfall pattern in the selected CMIP6 model simulations by eliminating all extreme rainfall events and testing the impact on dengue mortality. Here we considered extreme rainfall as those with values greater than the 90th percentile of the reference period rainfall time series (1995 and 2014). Our results show that the removal of extreme rainfall events lead to an increase in dengue mortality by about 3–4% (refer to Supplementary Figs. S4b, d, f, and S5). Thus, future extreme rainfall events can be expected to offset the increase in dengue by 3–4%. This result is consistent with our findings from observational data, which suggest that extreme rainfall tends to reduce dengue transmission, while moderate rainfall is likely to increase dengue incidence.

In summary, our study reveals that the projected increase in dengue mortality over Pune is primarily driven by the increase in temperature and changes in rainfall patterns. These results underscore the importance of a comprehensive and multivariate approach to assessing the risks associated with dengue transmission. Furthermore, it emphasizes the importance of reducing greenhouse gas emissions to mitigate the harmful impact of climate change on public health.

## Discussion

Dengue fever is a climate-sensitive vector-borne disease that is rapidly increasing worldwide, with significant spatial variability in its relationship with the climate drivers^1,62^. Mutheneni et al., which focus on the extrinsic incubation period (EIP, lifecycle of the virus inside the vector) and its variability in different climatic zones in India, argued that instead of a general model, future studies should focus on the development of forecasting models by climatic zones^62^. In India, most studies have focused on comprehending the epidemiological and entomological aspects of dengue, with limited information available on understanding the impacts of climate change on dengue transmission. The primary objective of the current study is to establish a model framework for region-specific insights into the influence of climate change on dengue occurrences. We investigated the climate-dengue links in Pune, an Indian district with a high dengue disease burden.

Incorporating the lag in climate-dengue associations provides early warnings with a sufficient lead time to prepare and respond effectively to dengue outbreaks. Also, it enables the use of real-time climatic variables for predictions. This can help reduce uncertainties associated with weather forecasts and improve the skill of predictions. Temperature, rainfall, and relative humidity are found to impact dengue mortality over Pune at a lag of five months, three months, and two months, respectively. This aligns with other studies that used dengue data with similar temporal resolution and observed lags exceeding four months between temperature changes and dengue incidence in rural Pune and other regions globally^63–65^. Although Aedes mosquitoes have a relatively short lifespan, the lag between the climate variables and dengue incidences need not be restricted to a single mosquito generation to remain biologically significant. Rather, the observable population fluctuations can arise from cumulative environmental changes across multiple mosquito generations^66^.

We employed machine learning to decipher the non-linear and cumulative impacts of these weather variables on dengue and developed a dengue prediction model as an early waning system for Pune. Our dengue model based on the random forest regression algorithm demonstrated good prediction skill (*r* = 0.77, NRMSE = 0.52) at a two-month lead time, accounting for the lag in the weather-dengue relationship. Further, the dengue model was used for the future projections of dengue mortality over Pune by utilizing climate projections from the selected CMIP6 models suited for the Indian monsoon region. To the best of our knowledge, this study is the first to employ machine learning and CMIP6 model outputs for regional future projections of a disease. Dengue future projection efforts in earlier studies do not consider the regional heterogeneity in climate-dengue associations, instead producing future projections for larger regions solely based on how the increase in global surface temperatures affect one or more epidemiological parameters^24,67^. Furthermore, the dengue future projections in earlier studies do not employ a model selection procedure based on the skill of models in representing the climate variability and change over the specific region under study.

Ensemble mean projections of weather variables from the selected CMIP6 models show that relative to the recent past (1995–2015), the mean surface temperature is likely to be higher by 1–3.5 °C, cumulative rainfall by 23–65 cm, and relative humidity by 0.4–1.6% in the future (2021–2100) under the three different SSPs considered (SSP1, SSP2, and SSP5) (refer Supplementary Table S2). In response to these climatic changes, dengue mortalities over Pune are projected to increase by 12–112% in the future (2021–2100) under low-to-high emission pathways. Our results align with the previous studies that reported that climate change can exacerbate vector-borne diseases^8,68^. The findings of this study have significant implications for policymakers, as they provide insights into the potential impacts of climate change on dengue mortality in Pune and provide a clear pathway to extend the model to any other region.

Our study has certain limitations. The current study uses dengue mortality data instead of dengue morbidity due to the unavailability of reliable dengue infection data. In India, dengue incidence data may be highly underreported due to issues such as irregular data management, inadequate filing of outbreak reports, and a tendency to under-report outbreaks due to fear of backlash from higher authorities^2,69–71^. Further research is needed to better understand the strengths and limitations of various types of dengue data sources. The use of mortality data instead of incidence data may introduce uncertainties in early warning systems and future projections of dengue. Also, all the lags considered in the current study are with respect to mortality and not morbidity. It is important to note that the lag between hospital admission and dengue mortality can vary by region due to factors such as the quality of healthcare services, predisposition to other health conditions, and comorbidities. However, quantifying these uncertainties is challenging without a study focused on dengue incidence for any given location. The current study considers dengue mortality as a proxy for dengue incidence in Pune based on two factors. First, the strong correlation between dengue incidence and mortality in India (*r* = 0.72, *p* < 0.05), as illustrated in Supplementary Fig. S6, suggests that the two variables are closely related. Second, the dengue mortality in Pune follows a seasonal pattern similar to that of dengue incidence, with a marked increase in mortality with the onset of monsoon rains with comparatively low mortality rates reported from December to May (Fig. 2a). These findings indicate that the variability in dengue incidence and mortality is closely aligned. The current dengue model developed for Pune can be improved with access to longer-term, reliable dengue incidence data. The value of incorporating long-term dengue incidence data to improve the early warning systems can hardly be overemphasized.

In addition, the current study is restricted to exploring the links between climatic factors and dengue mortality. We acknowledge that dengue mortality is influenced by a range of other factors, including socio-economic, demographic, and geographical variables, which are not included in our model. Their inclusion in future studies could further refine our understanding of the climate-dengue associations. Also, the future projections derived under the SSPs do not explicitly account for the localized evolution of factors such as socioeconomic conditions, urbanization, or the resilience and adaptive capacity specific to Pune. This has been a limitation in most existing studies on disease future projection that use climate projections from CMIP^72,73^. However, to improve the regional relevance of the climate change simulations used, the current study performed a model selection procedure to identify the CMIP6 models that better represent the climate characteristics of the study region.

Despite these limitations, the dengue model developed in this study demonstrates reasonable and usable forecast skill and the study provide a comprehensive framework for region-specific insights into the impact of climate on dengue occurrences. It also introduces a methodology for using climate change projections to assess future disease risks. Although this study focused specifically on dengue mortality in Pune, the overall framework of the study can be extended to any other region with dengue infections.

## Data

Pune, located in Maharashtra, India, covers an area of 15,643 sq. km (17°54′ to 19°24′ N, 73°29′ to 75°10′ E) and has a population of approximately 10 million (Fig. 2a,b). Pune is selected for the current study due to its high dengue disease burden as well as the region’s evident climate-dengue relationship, as demonstrated by previous studies^63,74,75^. The summer monsoon rainfall (June to September) has a significant impact on dengue occurrences in Pune, with more than 80% of dengue mortalities in the district occurring after the onset of the monsoon in June (Fig. 2c). This makes Pune an ideal study location to better understand the intricate rainfall-dengue relationship.

### Observed climate and health data

The current study uses dengue mortality data for Pune from 2001–2015, obtained from the health department of Pune Municipal Corporation (PMC). The dataset pertains to urban areas with 80% of the data reported from Pune city (Fig. 1, step 1). For the principal analyses in this study, we omitted the initial years (2001–2003) due to sparse data. To assess whether the dengue mortality over a region can represent its dengue incidences, we compared dengue cases and mortality from the National Center for Vector Borne Diseases Control (NCVBDC) data^3^. Our analysis reveals a statistically significant strong correlation (*r* = 0.72, *p* < 0.05) between dengue cases and mortality, demonstrating that dengue mortality can be used as a proxy for dengue morbidity (Supplementary Fig. S6).

To study the climate-dengue associations in Pune, the current study uses high-resolution daily gridded rainfall (0.25° × 0.25° grid)^76^ and temperature (1° × 1° grid)^77^ data from the India Meteorological Department (IMD), both validated using IMD station data at Pune. Relative humidity data, covering the same period as the health data (2004–2015), were obtained from the IMD’s Pune station (Fig. 1, step 2).

### Climate projections from CMIP6

CMIP6 offers the latest climate projections from multiple modeling centers worldwide under different socioeconomic and emission scenarios. The current study uses historical (1850–2015) and future (2015–2100) simulations from the CMIP6 models for the dengue future projection analysis. Future dengue projections under three distinct Shared Socioeconomic Pathways (SSPs), representing different levels of socioeconomic development and greenhouse gas emissions are investigated. The three pathways are: a world of sustainability-focused growth and equality where the radiative forcing is limited to 2.6 W m^−2^ by the end of the twenty-first century (SSP1-2.6, low emission pathway); a “middle of the road” world where trends broadly follow their historical patterns and the radiative forcing is limited to 4.5 W m^−2^ (SSP2-4.5, intermediate emission pathway); and the high road—a world of rapid and unconstrained fossil fuel-driven growth in economic output and energy use where the radiative forcing is high at 8.5 W m^−2^ (SSP5-8.5, high emission pathway)^78^. Fifteen CMIP6 models with all three predictor variables—temperature, rainfall, and relative humidity—available under SSP1, SSP2, and SSP5 are evaluated. Instead of a direct ensemble mean of the 15 models, models with a better skill in representing the Indian summer monsoon rainfall alone are selected for future projection analysis.

## Methods

### Climate based dengue model

To explore the non-linear and cumulative effects of selected climatic predictors on dengue mortality, we developed a machine learning model employing the Random Forest Regression (RFR) algorithm (Fig. 1, steps 4–12). The resulting dengue model, incorporating the lag in climate-dengue association, can function as a dengue early warning system for Pune with a two-month lead time (Fig. 1, steps 11 and 12). RFR is a machine learning ensemble method that combines several separately trained models to create a strong learner that can be used for classification and regression^47^. In this study, we implemented RFR using the Python package *scikit-learn*^79^.

The development of a dengue early warning model for Pune using the machine learning algorithm Random Forest Regression (RFR) involves many steps, such as the selection of best predictor variables, test-train split, optimization of model hyperparameters, and K-fold cross-validation. Various potential dengue predictors, such as rainfall, minimum temperature, maximum temperature, diurnal temperature range, relative humidity, specific humidity, vapour pressure, and wind speed were collected and analyzed to determine the climatic predictors of dengue mortality in Pune, and those contributed to the model performance alone were retained for predicting dengue mortality (Fig. 1, steps 2 and 3). Also, when variables were highly correlated (multicollinearity), only one was retained, as adding more did not enhance model skill. The Pearson correlation and cross-correlation analyses were performed to aid in the selection of the best climatic predictors of dengue and to identify the lag in climate-dengue associations in Pune (Fig. 1, step 3). The final dengue model is obtained after the following procedures:

#### Train-test data split

To develop a machine learning model, both the predictors and target are randomly divided into two parts: the training data for learning and test data for testing the model (Fig. 1, step 4). The importance of the train-test split is that the training set contains known output from which the model learns, and the test set then tests the model’s performance. For better predictions, a significant portion of the dataset is used for training. Here, we have used the data from 2005 to 2014 for training the model and the years 2004 and 2015 for testing. The years 2004 and 2015 were chosen for testing as 2004 is a year reporting low dengue mortality, and 2015 is the recent year with high dengue mortalities. Consequently, if the model can effectively predict outcomes for these two contrasting years, it demonstrates model robustness.

#### Model hyperparameter optimization

Hyperparameter tuning was done to improve model performance, in which the optimal hyperparameters for each problem were selected (Fig. 1, step 6). RFR algorithm has many parameters, such as n_estimators, max_features, max_depth, and min_sample_split^80^. For an effective model, their values must be carefully selected^81^. This is normally done by running an optimization procedure that selects parameters that minimize prediction error. In this study, the best model hyperparameters were found using RandomSearchCV, a module from the machine-learning Python library *Scikit-learn*^79^.

#### K-fold cross-validation (CV)

K-fold cross-validation is a method used to validate models while tuning hyperparameters in machine learning (Fig. 1, step 7). Multiple rounds (k) of validation were performed using different partitions of a dataset, and the result was averaged over the ‘k’ rounds to evaluate the model performance.

We used Normalized Root Mean Square Error (NRMSE) and Pearson correlation coefficient to evaluate the goodness of the model fit. NRMSE is a metric that measures the goodness of model fit in relation to the spread or variability of the data. A lower NRMSE and a higher correlation coefficient value indicate better model performance. Further, to gain insights into the impact of various input weather variables on the predictions given by the dengue model, we utilized the variable importance feature of the Random Forest algorithms. Variable importance refers to the degree of association between a given predictor variable and the model predictions. By evaluating the variable importance of input features, we were able to determine their significance in predicting dengue mortality in Pune.

### Dengue early warning system and future projections for Pune

The dengue model developed based on observed climate-dengue associations in Pune can serve as an early warning system with real-time inputs (Fig. 1, steps 11 and 12) and can also be used to prepare future dengue projections using climate change projections from CMIP6 models (Fig. 1, steps 13 and 14). To obtain dengue mortality predictions for Pune, selected climatic predictors at their respective optimal lags from real-time observations are used as inputs. For future dengue projections, future climate simulations (2015–2100) from selected CMIP6 models are provided as inputs to the dengue model. Incorporating the lag in the climate-dengue association enables dengue mortality predictions two months ahead, providing sufficient lead time for an early warning forecast targeted at curbing dengue outbreaks.

We assessed the fidelity of CMIP6 models in representing the rainfall climatology and its intraseasonal oscillations by comparing model historical rainfall data (1980–2014) over the Indian region (70° E–100° E, 10° N–30° N) with the IMD gridded rainfall data (1980–2014) using the following two criteria. The first criteria checks if the June to September mean rainfall of the model shows a significant pattern correlation with that of the observations and thus selects models with a better spatial representation of the mean Indian summer monsoon rainfall. The second criteria use the standard deviation of summer monsoon rainfall data filtered for 30–90 days to evaluate how well the model represents the year-to-year changes in the monsoon intraseasonal oscillations. The pattern correlation coefficient between the standard deviations of the 30–90 days filtered rainfall from the observations and model simulations is calculated for the Indian region, and models with a statistically significant correlation alone are considered for further analysis (Supplementary Fig. S2).

Eight models that satisfy the above two criteria are selected for further analysis (Supplementary Table 1). Since we are using the ensemble mean of the model projections and most of the models have sufficiently high resolutions (100 km), no additional downscaling is done. The selected model outputs are interpolated into a common spatial grid (1° × 1°) by bilinear interpolation. Bias correction using the quantile mapping method is applied to the selected CMIP6 models^82^. Quantile mapping is a nonparametric method that matches the full distribution variable values between the model and observations by adjusting the mean and variance of a model simulation to agree with the statistical properties of the observations^83^. After bias correction, a one-degree box around Pune is considered for analyzing the future projections.

We conducted several model experiments to understand the impacts of future climate change on dengue mortality in Pune. Climate predictors from the selected CMIP6 models were detrended individually while keeping other predictors unchanged, and the corresponding impact on future dengue projections was studied. In addition, we modified the rainfall patterns in the CMIP6 simulations by removing all extreme rainfall events to evaluate their relative impact on dengue mortality.

## Supplementary Information

Supplementary Information.

## Data Availability

The dengue mortality data for Pune were obtained from the health department of Pune Municipal Corporation (PMC) under a data agreement and can be accessed via request submitted to the PMC. Researchers can refer to the corresponding author for more information on accessing the data. Observed meteorological data is available in the public domain from the India Meteorological Department, and the CMIP6 model outputs are available through the CMIP6 data archive developed and operated by the Earth System Grid Federation (https://esgf-node.llnl.gov/search/cmip6/). The code sources are available from the corresponding author upon request.
