# Supplementary material for: Dengue dynamics, predictions, and future increase under changing monsoon climate in India
Source: Sci Rep. 2025 Jan 21;15:1637. doi: 10.1038/s41598-025-85437-w (PMC11750985; doi:10.1038/s41598-025-85437-w)
Supplement: Supplementary file 1 — Supplementary Information. [file 41598_2025_85437_MOESM1_ESM.docx]

**Supplementary Information**

**Dengue Dynamics, Predictions, and Future Increase under Changing Monsoon Climate in India**

Sophia Yacob, M. K. Roxy, Raghu Murtugudde, Anand Karipot, Amir Sapkota, Panini Dasgupta, Kalpana Baliwant, Sujata Saunik, Abhiyant Tiwari, Rajib Chattopadhyay, Revati K. Phalkey

**Contents**

**Table S1: CMIP6 models selected for the current study and their spatial resolution 2**

**Table S2: Ensemble mean projections of climate-based dengue predictors 3**

**Table S3: Correlation between cumulative rainfall of monsoon months and dengue mortality 4**

**Table S4: Future trends in meteorological predictors of dengue mortality 5**

**Figure S1: Climate-based dengue metric for Pune 6**

**Figure S2: Selection of CMIP6 models for future projection analysis 7**

**Figure S3: Future projections of climatic predictors of dengue mortality in Pune 8**

**Figure S4: Future projections of dengue mortality in Pune after detrending experiment 9**

**Figure S5: Percentage change in dengue mortality after detrending experiment 10**

**Figure S6: Comparison between reported dengue incidences and mortality 11**

**Figure S7: Cumulative rainfall maps for monsoon months 12**

| **Model** | **Resolution (km)** |
| --- | --- |
| CESM2-WACCM | 100 |
| CMCC-CM2-SR5 | 100 |
| CMCC-ESM2 | 100 |
| MIROC6 | 250 |
| MPI-ESM1-2-LR | 250 |
| NorESM2-LM | 250 |
| NorESM2-MM | 100 |
| TaiESM1 | 100 |

**Table S1.** CMIP6 models selected for the current study and their spatial resolution. These models best represent the spatial distribution of mean rainfall and interannual variability in the intraseasonal oscillations of the Indian summer monsoon in the historical simulations from 1980 to 2014.

| **SSP1** | Temperature (°C) | Rainfall (mm) | Relative humidity (%) | Active days | Break days |
| --- | --- | --- | --- | --- | --- |
| Reference Period | 26 | 2109 | 81.87 | 34 | 36 |
| Near future | 26.59 | 2363 | 82.5 | 35 | 38 |
| Mid-century | 27.07 | 2375 | 82.19 | 36 | 39 |
| Late century | 27.25 | 2394 | 82.07 | 36 | 39 |

| **SSP2** | Temperature (°C) | Rainfall (mm) | Relative humidity (%) | Active days | Break days |
| --- | --- | --- | --- | --- | --- |
| Near future | 26.62 | 2221 | 82.53 | 35 | 39 |
| Mid-century | 27.13 | 2346 | 83.1 | 37 | 39 |
| Late century | 28 | 2465 | 82.66 | 37 | 41 |

| **SSP5** | Temperature (°C) | Rainfall (mm) | Relative humidity (%) | Active days | Break days |
| --- | --- | --- | --- | --- | --- |
| Near future | 26.61 | 2271 | 82.88 | 35 | 36 |
| Mid-century | 27.56 | 2384 | 82.79 | 36 | 38 |
| Late century | 29.49 | 2760 | 83.40 | 37 | 42 |

**Table S2.** Ensemble mean projections of temperature, rainfall, relative humidity, active and break days of monsoon from the selected eight CMIP6 models. Projections for the reference period (1995–2014), near future (2021–2040), mid-century (2041–2060), and late century (2081–2100) are presented in the table under the Shared Socioeconomic Pathways, SSP1 (sustainability pathway, low emission scenario), SSP2 (middle-of-the-road pathway, intermediate emission scenario), and SSP5 (fossil-fuel intensive pathway, high emission scenario).

| **Month** | **Pearson correlation coefficient** |
| --- | --- |
| June | -0.66 (*p*=0.01) * |
| July | -0.21 (*p*=0.20) |
| August | -0.20 (*p*=0.20) |
| September | -0.01 (*p*=0.31) |

**Table S3:** Pearson correlation coefficient between annual dengue mortality and monthly cumulative rainfall of monsoon months (June to September) from 2004–2015 in Pune. Cumulative rainfall of June shows a statistically significant (denoted by *) negative correlation with dengue mortality in Pune.

| **Variables** | **Trend/century** | | |
| --- | --- | --- | --- |
|  | SSP1-2.6 | SSP2-4.5 | SSP5-8.5 |
| **Temperature (°C/century)** | 1.26* | 2.3* | 4.6* |
| **Rainfall (mm/century)** | 1.61* | 8.7* | 15.3* |
| **Relative humidity (%/century)** | -0.3 | 0.2 | 1.06* |
| **Active days (days/century)** | 1.5 | 3.8* | 7.03* |
| **Break days (days/century)** | 1.1 | 3.2* | 9.01* |

**Table S4.** Future trends (per century) in meteorological predictors of dengue mortality in Pune from 2015 to 2100. Trend values are based on the ensemble mean of eight selected CMIP6 models. Statistically significant trends (*p* < 0.05) are marked with an asterisk (*).


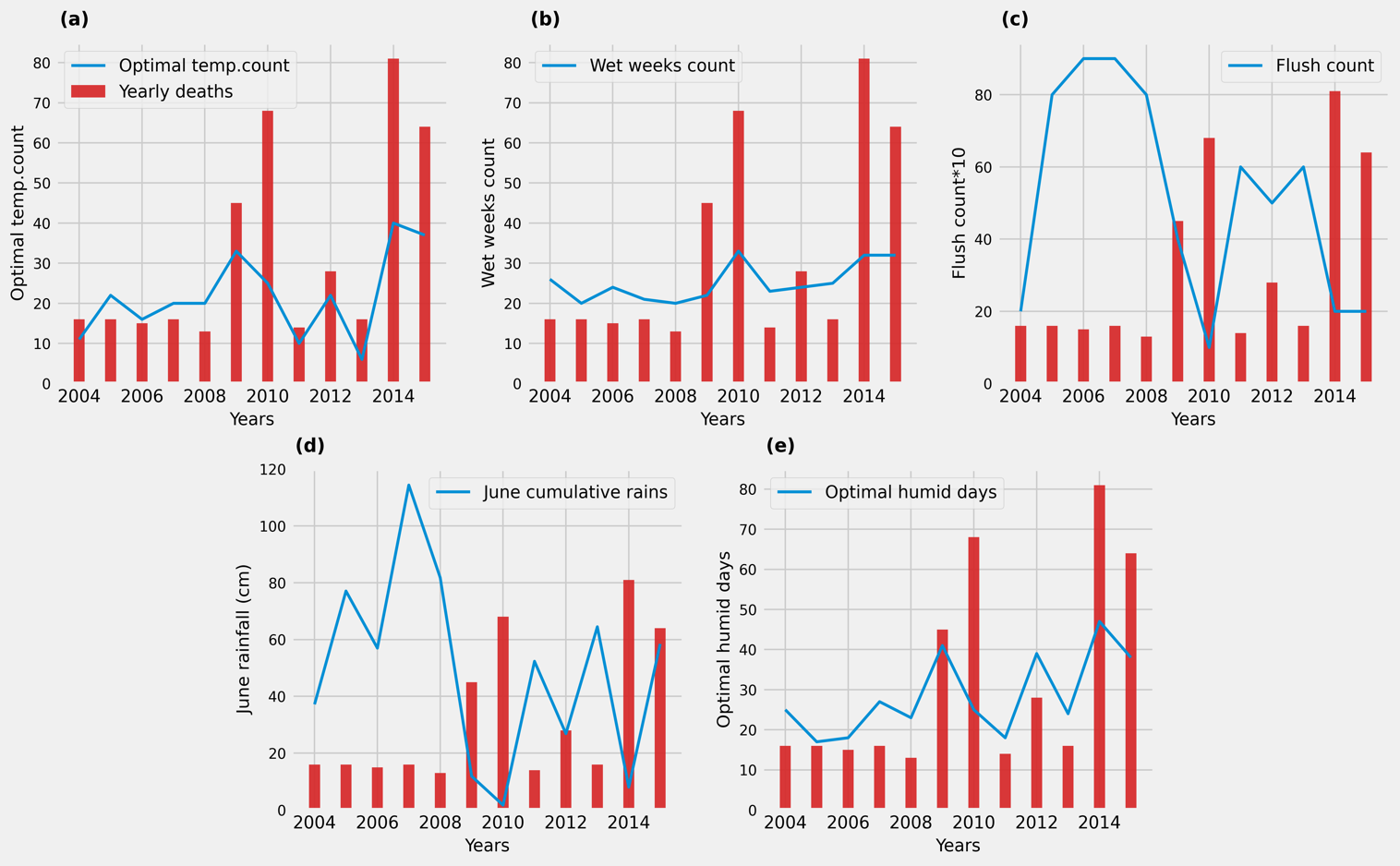


**Figure S1.** Climate-based dengue metric for Pune: (a) count of days with optimal temperature, (b) count of wet weeks, (c) count of flush events, (d) June cumulative rains, and (e) count of days with optimal relative humidity. Red bars represent annual dengue mortalities, and blue lines indicate meteorological predictor variables from 2004 to 2015.


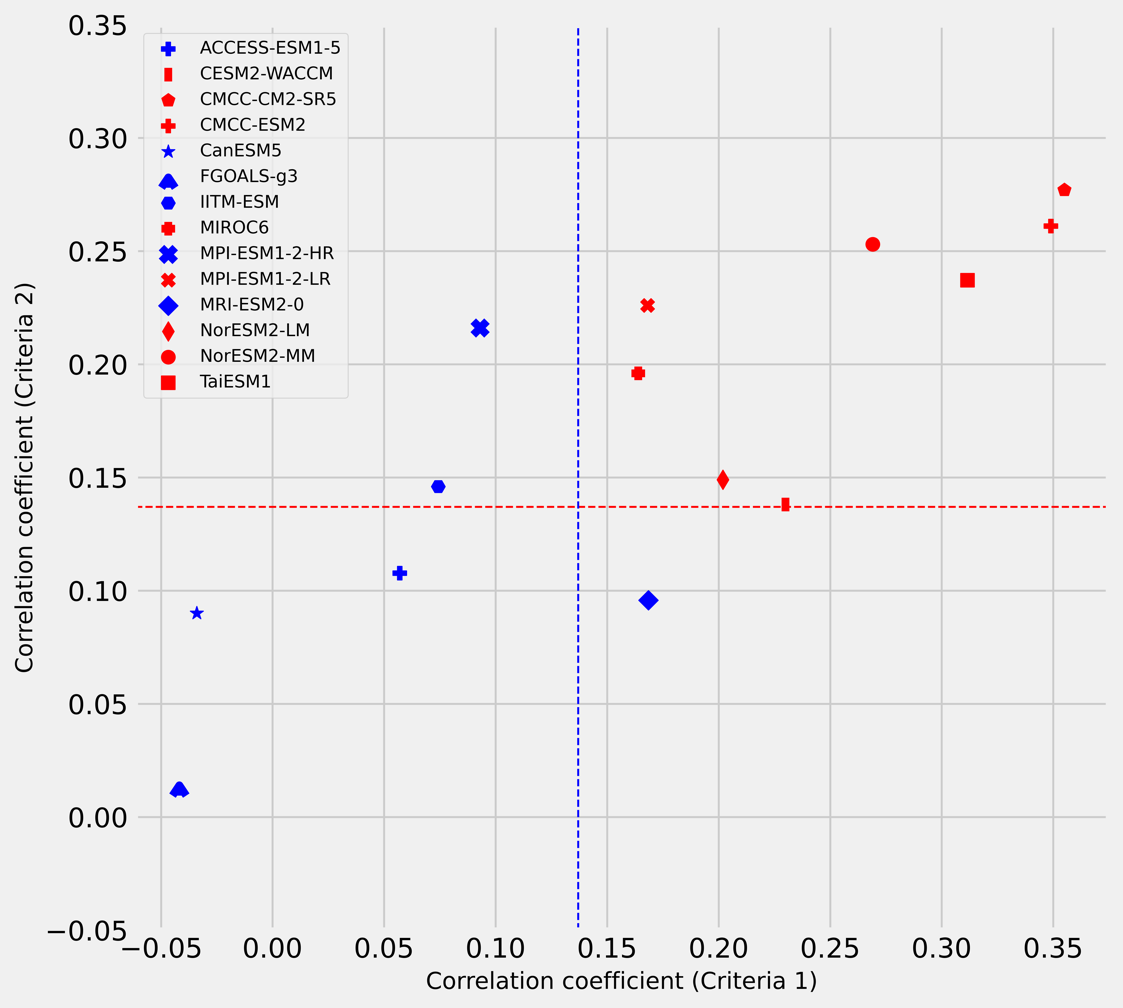


**Figure S2**. The figure illustrates the pattern correlation coefficient between observations and CMIP6 models across the Indian region (70° E–100° E, 10° N–30° N), focusing on two criteria: criteria 1, representing mean rainfall, and criteria 2, depicting the interannual variability (standard deviations) in the 30–90 days oscillations of the Indian summer monsoon rainfall for the period 1980–2014. Dashed red and blue lines indicate the threshold values above which the pattern correlations are statistically significant (*p* < 0.05) for criteria 1 and 2, respectively. Red markers highlight selected models that satisfy two criteria with a statistically significant pattern correlation coefficient.


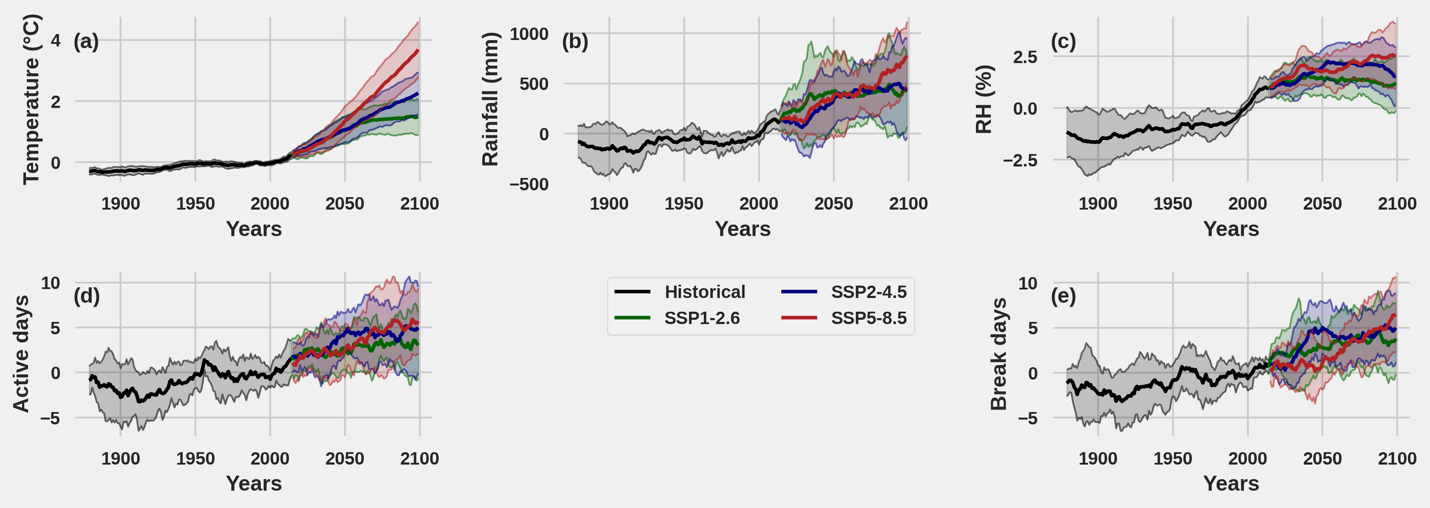


**Figure S3.** The multi-model ensemble mean of meteorological predictors of dengue mortality over Pune for the historical time period (1860–2014) (black line) and for the future (2015–2100), relative to the reference period mean (1995–2014) under the Shared Socioeconomic Pathways, SSP1 (green line, sustainability pathway with low emission scenario), SSP2 (blue line, middle-of-the-road pathway with intermediate emission scenario), and SSP5 (red line, fossil-fuel intensive pathway with high emission scenario). The 20-year smoothed time series from the selected CMIP6 models were used to calculate the multi-model ensemble mean of (a) mean temperature, (b) cumulative rainfall, (c) mean relative humidity, (d) count of active days, and (e) count of break days. Shading in the time series represents the range of mean plus/minus one standard deviation of the model spread.


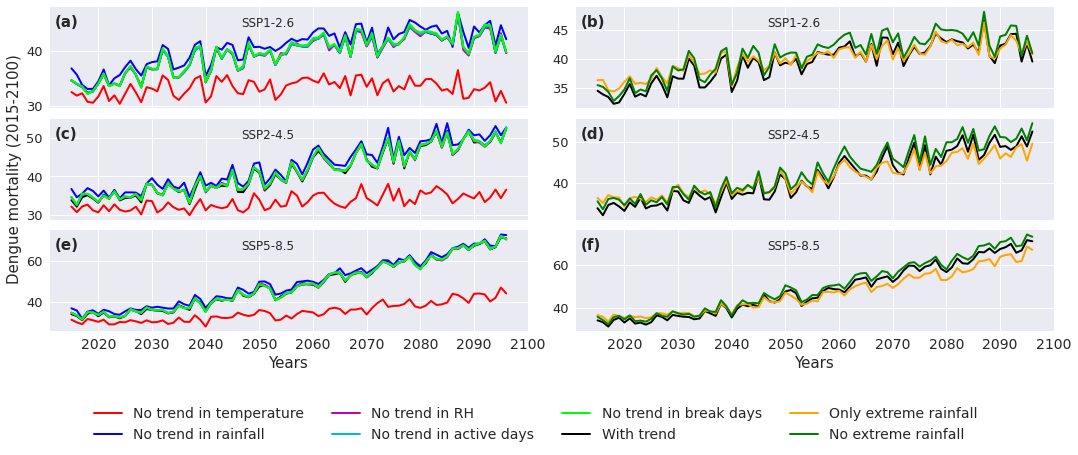


**Figure S4.** Annual dengue mortality projections for Pune (2015–2100) after making changes in the climatic predictors under the emission scenarios (a) and (b) SSP1 (sustainability pathway with low emission scenario), (c) and (d) SSP2 (middle-of-the-road pathway with intermediate emission scenario), and (e) and (f) SSP5 (fossil-fuel intensive pathway with high emission scenario). Results from the following changes in the climatic predictors from the selected CMIP6 models are shown: detrending temperature (red line), rainfall (blue line), relative humidity (magenta line), active days (cyan line), break days (lime line), and changing rainfall pattern by removing all extreme rainfall (dark green) and keeping only extreme rainfall (orange). Extreme rainfall considered here are those above the 90^th^ percentile of the reference period rainfall (1995–2014). The black line depicts the original dengue mortality projections from the dengue model without changes in climatic predictors.


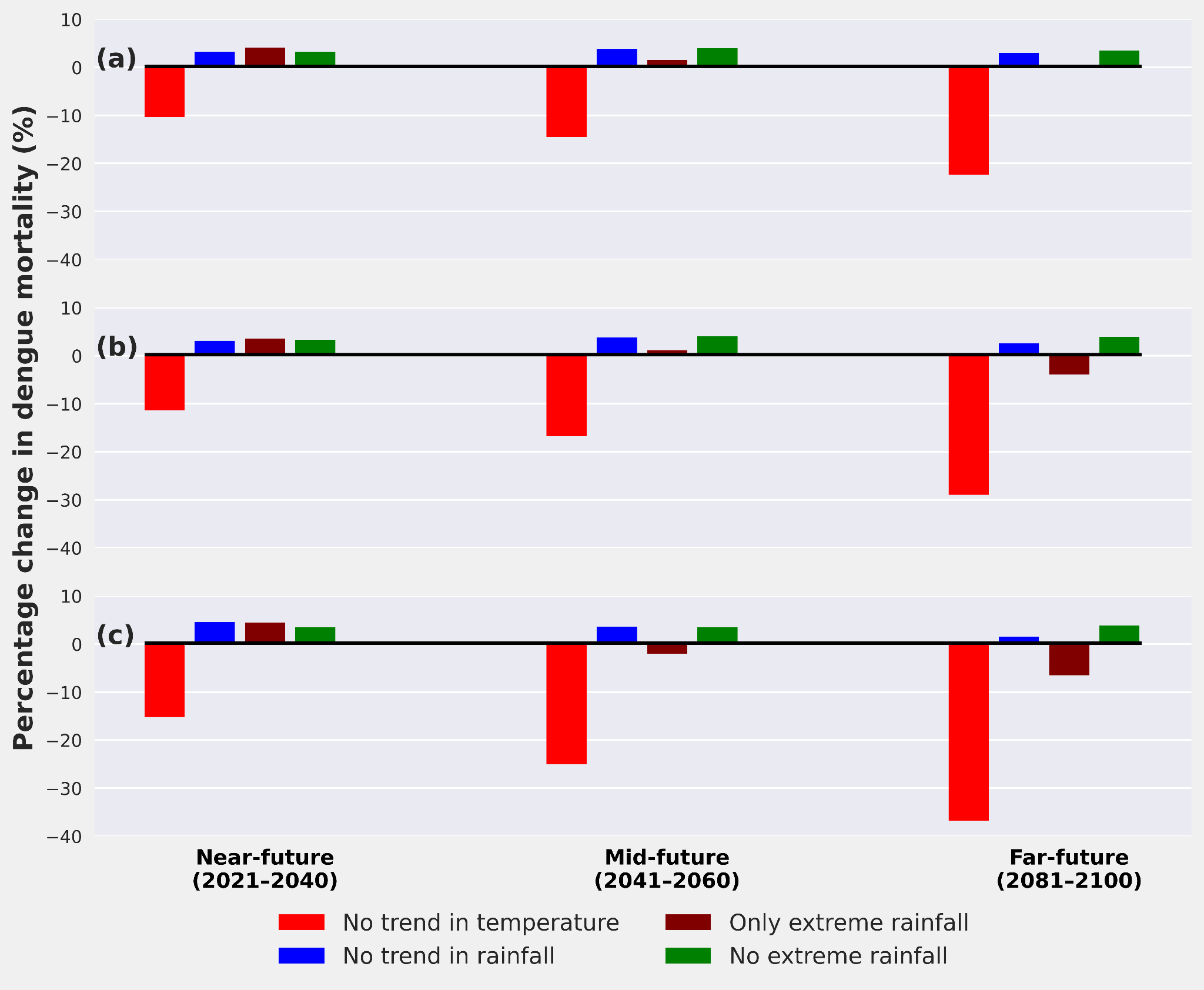


**Figure S5.** Percentage change in dengue mortality projections in Pune, after making changes in the climatic predictors under the future Shared Socioeconomic Pathways (SSPs), (a) SSP1 (sustainability pathway with low emission scenario), (b) SSP2 (middle-of-the-road pathway with intermediate emission scenario), and (c) SSP5 (fossil-fuel intensive pathway with high emission scenario) for the near future (2021–2040), mid-century (2041–2060), and late century (2081–2100). The changes include removing the trend in temperature (red bar) and trend in rainfall (blue bar), keeping only extreme rainfall (maroon bar), and removing all extreme rainfall (green bar) from the selected CMIP6 model outputs. Extreme rains considered here are rainfall above the 90^th^ percentile of the reference period (1995–2014). The percentage change shown is relative to the dengue mortality projections with no change in climatic predictors.


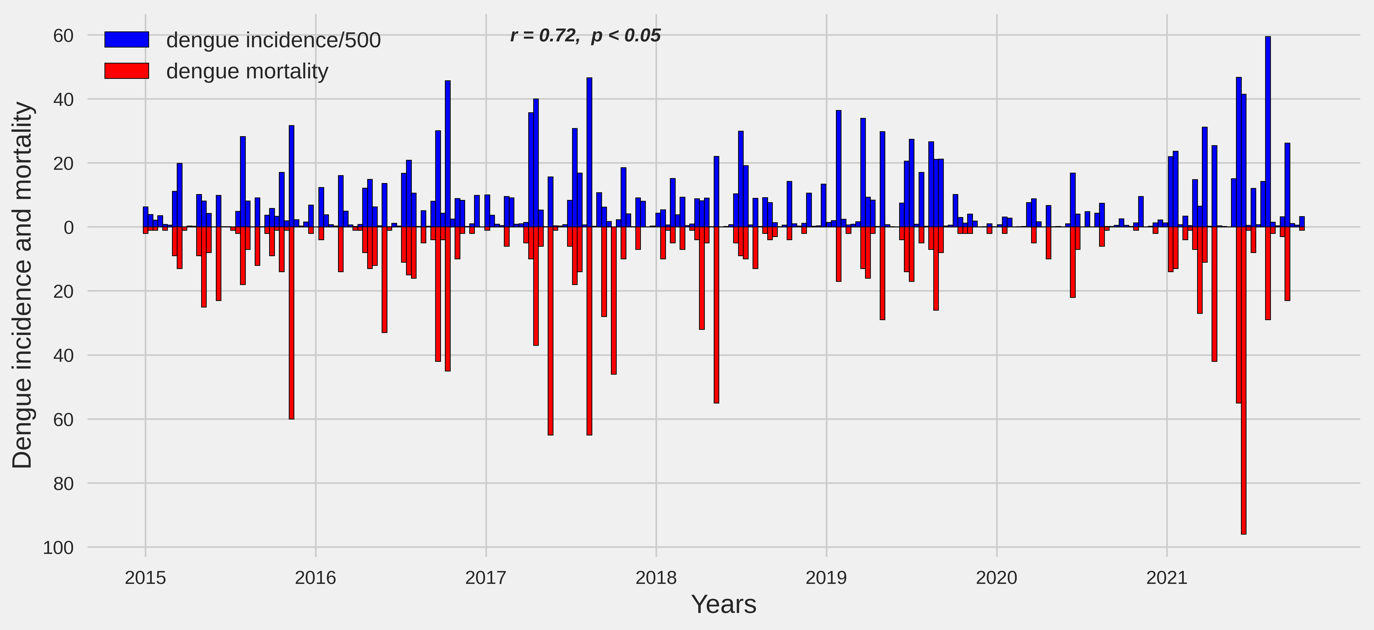


**Figure S6.** Comparison between reported dengue incidences (blue bars) and mortalities (red bars) across thirty-five states and union territories in India from 2015 to 2021, except for West Bengal and Lakshadweep, due to incomplete data. Each blue and red bar in the plot represents the dengue incidence and mortality, respectively, in a state/union territory for a particular year. The analysis, based on data from the National Center for Vector Borne Diseases Control, reveals a strong positive correlation (*r* = 0.72, *p* < 0.05) between dengue incidence and mortality, demonstrating that dengue mortality can be used as a proxy for dengue incidence.

**Figure S7.** Cumulative rainfall maps for the monsoon months from June to September in Pune. Black dotted line indicates annual dengue mortality reported in Pune during the study period (2004–2015).
